# The Impact of Various Natural Gas Contaminant Exposures on CO_2_/CH_4_ Separation by a Polyimide Membrane

**DOI:** 10.3390/membranes10110324

**Published:** 2020-10-31

**Authors:** Nándor Nemestóthy, Péter Bakonyi, Piroska Lajtai-Szabó, Katalin Bélafi-Bakó

**Affiliations:** Research Group on Bioengineering, Membrane Technology and Energetics, University of Pannonia, 8200 Veszprém, Hungary; nemesn@almos.uni-pannon.hu (N.N.); bakonyip@almos.uni-pannon.hu (P.B.); tpiroska94@gmail.com (P.L.-S.)

**Keywords:** gas separation, polyimide membrane, natural gas separation, pollutant effects, stability measurements

## Abstract

In this study, hollow fibers of commercial polyimide were arranged into membrane modules to test their capacity and performance towards natural gas processing. Particularly, the membranes were characterized for CO_2_/CH_4_ separation with and without exposure to some naturally occurring contaminants of natural gases, namely hydrogen sulfide, dodecane, and the mixture of aromatic hydrocarbons (benzene, toluene, xylene), referred to as BTX. Gas permeation experiments were conducted to assess the changes in the permeability of CO_2_ and CH_4_ and related separation selectivity. Compared to the properties determined for the pristine polyimide membranes, all the above pollutants (depending on their concentrations and the ensured contact time with the membrane) affected the permeability of gases, while the impact of various exposures on CO_2_/CH_4_ selectivity seemed to be complex and case-specific. Overall, it was found that the minor impurities in the natural gas could have a notable influence and should therefore be considered from an operational stability viewpoint of the membrane separation process.

## 1. Introduction

The applicability of membranes in the processing of natural gas has been shown widely [1]. As the composition of (raw) natural gas varies considerably in line with its source, there are numerous tasks for improving its quality (e.g., methane content) and to meet pipeline and utilization requirements [2]. In fact, gas separation membranes (alone or in combination with other systems) can contribute to major technological steps, such as the removal of CO_2_, acidic components (particularly H_2_S), longer-chain hydrocarbons, water (vapor), and N_2_ [3]. To get the actual job done, gas separation membranes manufactured with the use of polymers gained broad recognition at laboratories, as well as on an industrial scale, and among the available materials, the glassy-polymer polyimide (commonly in hollow-fiber membrane modules) is one of the most well-known [4,5].

Polyimide is characterized by good CO_2_ permeability and simultaneous retention of CH_4_, resulting in sufficiently high CO_2_/CH_4_ selectivity [6]. However, a larger quantity of CO_2_ can make the membrane materials, including polyimide, suffer from plasticization, especially under higher feed pressure conditions [7]. As this penetrant-induced plasticization phenomenon (occurring in the presence of more notably condensable, soluble molecules) undermines the sensitive balance between the productivity of the separation (reflected in the permeability) and the purity of the product (influenced by the selectivity) [8], actions are still needed to design and synthetize better derivatives of polymers (with enhanced resistance to plasticization) via approaches such as blending and chemical crosslinking, etc. [9]. Still, choices and decisions are frequently needed as to whether the component permeability or the separation selectivity is more important in the given situation [10]. The dilemma of this trade-off has been addressed and assessed in depth by different studies based on the upper-bound relationship [11,12]. Moreover, the polyimide, and in general the glassy polymers, can be prone to physical aging, which may appear as a drawback in the long term due to the decrease of achievable gas fluxes [13,14].

Besides the issues related to carbon dioxide, other accompanying impurities may also cause adverse effects and deteriorate the performance of the membrane unit. Among the aforementioned components, the aggressive compound, the hydrogen sulfide is also regarded as a plasticizing agent using glassy polymers. From mixed gas permeation measurement applying ternary CH_4_/CO_2_/H_2_S, the relatively faster transportation of H_2_S through polyimide was concluded and is beneficial for its removal [15]. Such a step, the removal of acidic substances from the natural gas, is also referred to as the “sweetening” [16]. Interestingly, a recent paper using polyimide membranes reported the unexpected advantage of plasticization in H_2_S/CH_4_ separation, thanks to enhanced sorption coefficient [17]. However, at the same time, in agreement with common literature observations, the plasticization depressed the separation efficiency for the CO_2_/CH_4_ gas pair. As a matter of fact, there might be a necessity to develop process configurations, where the separations of H_2_S/CH_4_ and CO_2_/CH_4_ are carried out in the cascade of different, appropriately selected membranes [18]. Furthermore, removal of hydrocarbons (mainly C_3_+) from the raw natural gas should be taken into consideration [19]. The paraffin and olefin components have a higher commercial value and thus, their recovery is an economic interest. Additionally, the contact of aromatic hydrocarbons (containing the benzene-ring, e.g., toluene) and polyimide membranes was shown to affect membrane separation performance and the attainable CO_2_/CH_4_ separation selectivity [20].

In this work, we present the results of our study conducted on commercial (UBE Industries, LTD.) polyimide membrane fibers in a single-gas experimental permeation apparatus and comparatively evaluate the impacts linked to various exposures of H_2_S, dodecane hydrocarbon, and a mixture of benzene, toluene, and xylene (BTX) on CO_2_ and CH_4_ permeability and CO_2_/CH_4_ selectivity. The aim of this work is to deliver some new insights to the behavior of polyimide gas separation membranes under conditions when impurities (that are typically contained by the natural gas) are present during the separation of methane from carbon dioxide.

## 2. Materials and Methods

In this work, the effect of pollutants on the permeability of carbon dioxide (99.5%) and methane (99.95%) was investigated. The examined pollutants were H_2_S, a benzene-toluene-xylene mixture in a 1:1:1 ratio called BTX and n-dodecane. H_2_S was generated, as already mentioned in our earlier paper [21], and diluted thereafter with nitrogen (99.995%) to adjust the required concentration (Table 1). Every gas (CO_2_, CH_4_, N_2_) was used from a cylinder (Messer Hungarogáz Kft., Veszprém, Hungary). N-dodecane (98.0%) was provided by Sigma–Aldrich (Taufkirchen, Germany), benzene (99.5%) by Spektrum-3D Kft. (Debrecen, Hungary), toluene (99.8%) by Merck KGaA (Darmstadt, Germany), and xylene (98.5%) by Sigma–Aldrich (Taufkirchen, Germany).

**Figure 1:**
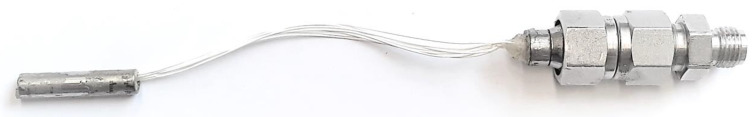
The membrane module containing the polyimide capillaries.

**Figure 2:**
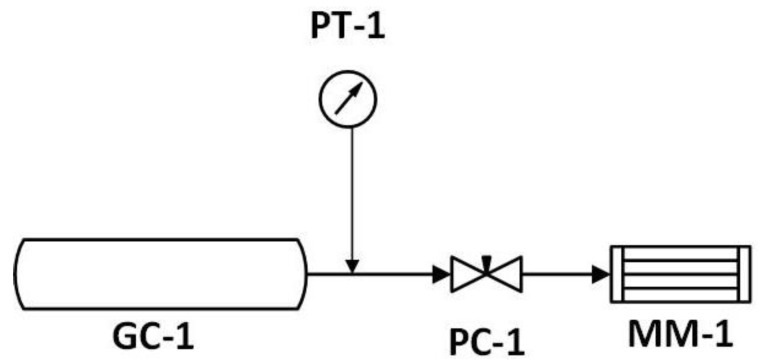
The layout of the experimental gas permeation apparatus.

For the experiments, polyimide capillaries were taken from a hollow fiber gas separation membrane (synthesised by UBE). A module consisted of six capillaries, for which ends were closed to get a “sack” (dead-end) configuration (Figure 1). The scheme of the gas separation test system can be seen in Figure 2. The actual test gas was filled to the gas container (GC-1), the pressure of which was monitored by a digital (WIKA A-10 type) pressure transducer (PT-1). During the measurements, the feed pressure of the membrane module (MM-1) was regulated and fixed by valve PC-1.

Gas permeability measurements were carried out according to the constant pressure (CP) method [22] with a constant volume (CV) [23] pressure chamber. The amount of the permeated gas was calculated by the CV method [24] (Equation (1)):

(1) $$n=\frac{P_{pc}\cdot V}{R\cdot T}$$

where R is the gas universal constant, T is the temperature (K), and V is the volume of the gas chamber (m^3^). P_pc_ is the pressure change in the chamber (Pa). The gas permeability coefficient, P, can be given by Equation (2):

(2) $$P=\frac{n\cdot L}{A\cdot t\cdot P_{d}}$$

where L (m) is the membrane thickness, A (m^2^) is the area of the membrane for gas permeation, and P_d_ is the pressure difference (Pa) across the membrane. The P could be converted to the unit of Barrer (1 Barrer = 3.35 × 10^−16^ mol·m^−1^·s^−1^·Pa^−1^).

**Figure 3:**
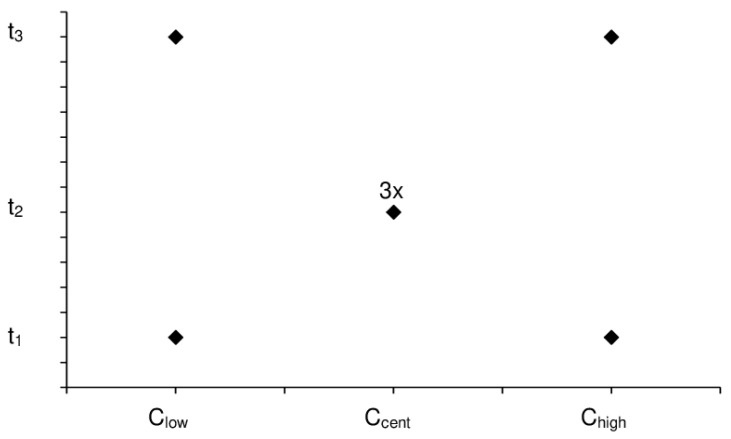
The layout of the experimental plan carried out in this study considering the conditions in Table 1.

**Figure 4:**
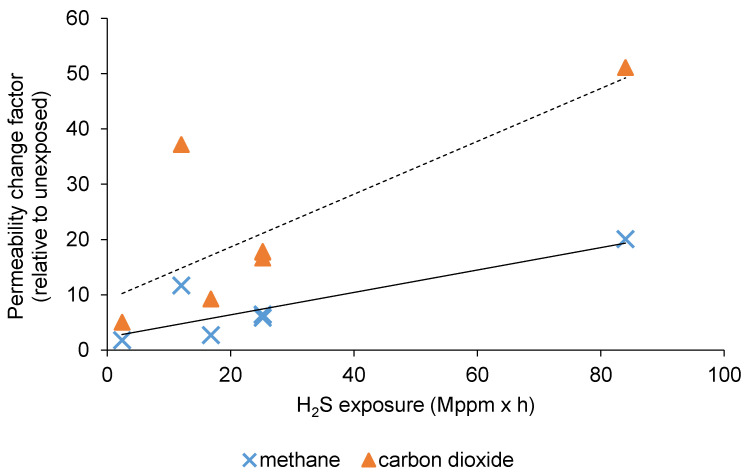
Alteration of gas permeabilities after exposures to H_2_S (the dotted trend line belongs to CO_2_).

**Figure 5:**
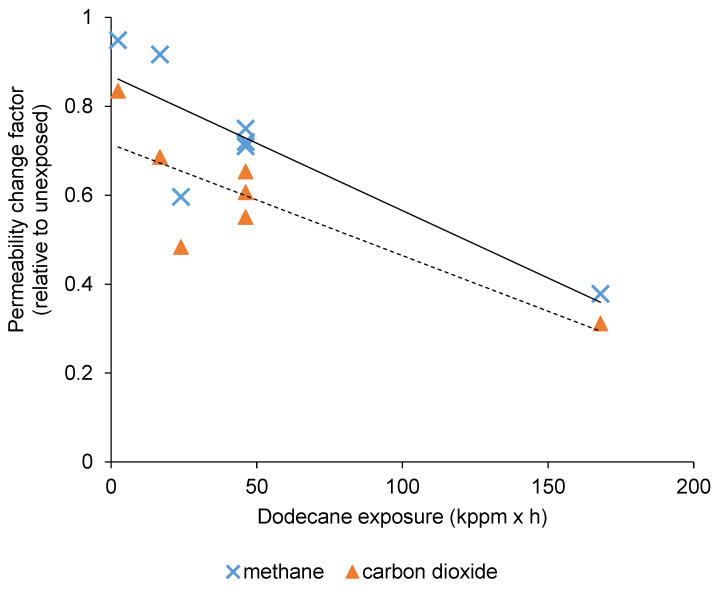
Alteration of gas permeabilities after exposures to dodecane (the dotted trend line belongs to CO_2_).

**Figure 6:**
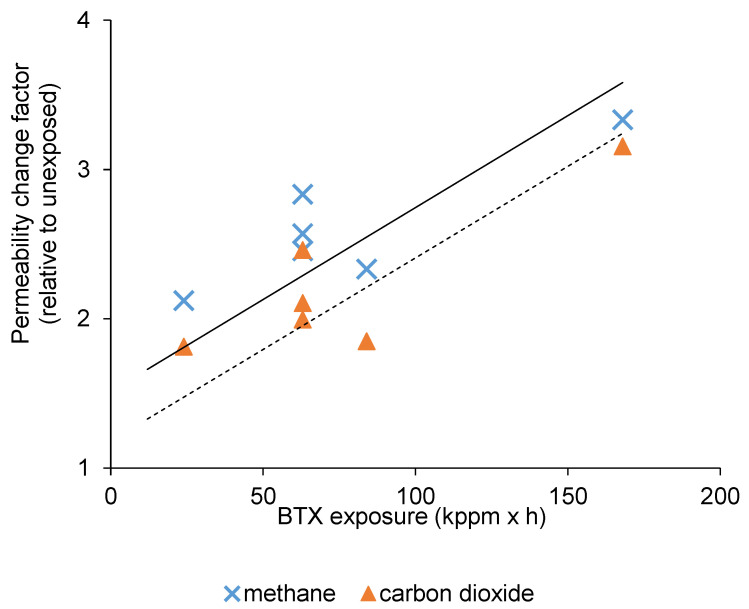
Alteration of gas permeabilities after exposures to benzene, toluene, and xylene (BTX) (the dotted trend line belongs to CO_2_).

**Figure 7:**
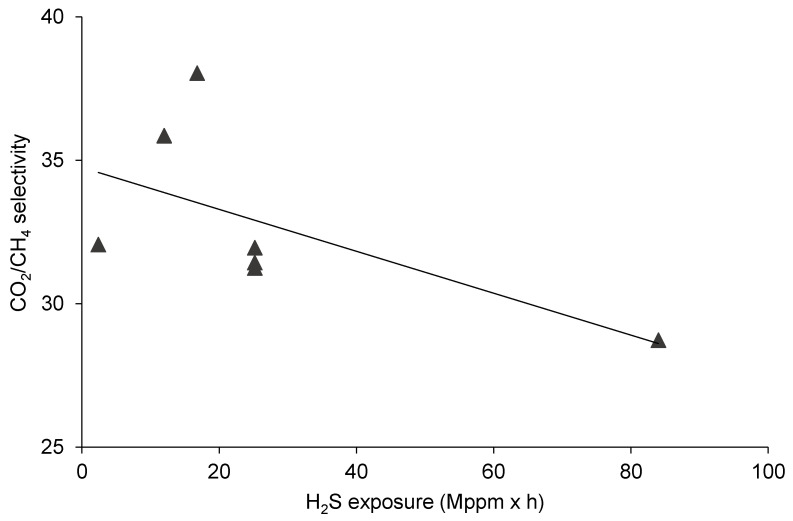
The effect of H_2_S exposure on CO_2_/CH_4_ selectivity.

**Figure 8:**
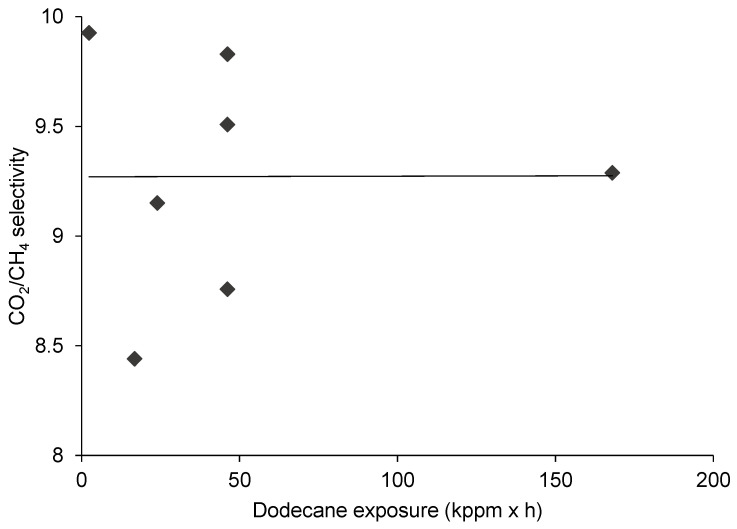
The effect of dodecane exposure on CO_2_/CH_4_ selectivity.

**Figure 9:**
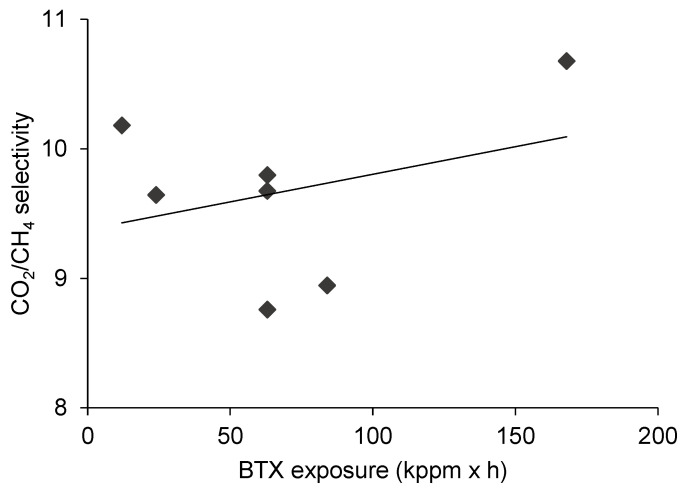
The effect of BTX exposure on CO_2_/CH_4_ selectivity.

To investigate the pollutant’s effect, the membranes were put in a closed vessel for a given time (t_1_, t_2_, t_3_), for which headspace contained a certain concentration of the given pollutant (C_min_, C_cent_, C_max_), as displayed by Table 1**,** where it can be noticed that the concentration boundaries within a particular case were equally-spaced. The C_cent_ was repeated three times (to check the confidence of the measurements under fixed conditions), and a total number of seven data points with fairly balanced distributions could be considered in all cases, according to Figure 3. The vessels were incubated at constant temperature (27 °C). The permeability of every membrane module was measured before the experiments (pristine polyimide) and directly after the desired incubation, and then, a permeability change factor (Figure 4, Figure 5 and Figure 6) was calculated as the ratio of respective gas permeabilities measured on the exposed and unexposed polyimide membranes. The parameter called exposure (the pollutant concentration multiplied by the time) was used as an independent variable to characterize the effects of pollutants on gas permeation (Figure 4, Figure 5 and Figure 6) and separation selectivity behavior (Figure 7, Figure 8 and Figure 9). For example, if the membrane is exposed to 1000 ppm of pollutant for 0.1 h, the exposure is equal to 100 ppm × h. The BTX and dodecane concentrations in Table 1 were estimated by the Antoine equation in CHEMCAD [25].

## 3. Results and Discussion

**Figure 10:**
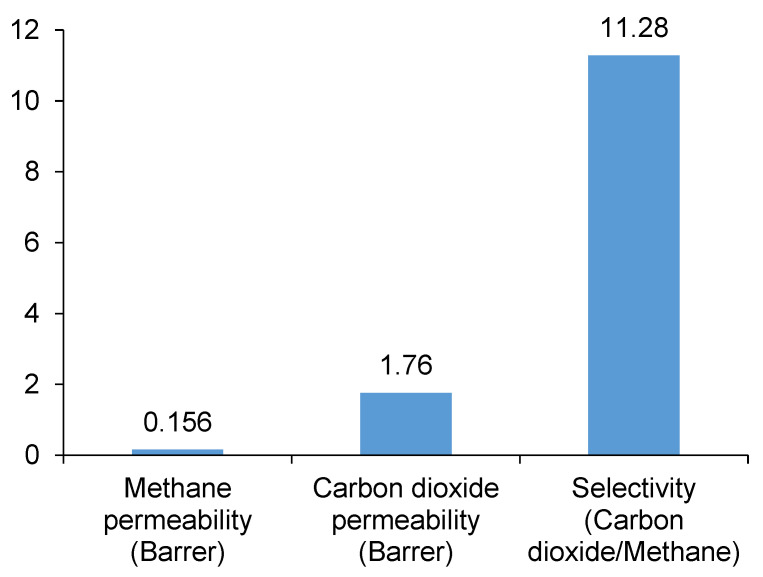
Permeation traits of CO_2_ and CH_4_ across fresh polyimide membrane.

First, the permeation of pure CO_2_ and CH_4_ gases was examined using the membrane prepared using the pristine polyimide hollow fibers. According to Figure 10, there was one order of magnitude difference in terms of the permeabilities: 0.156 Barrer and 1.76 Barrer for carbon dioxide and methane, respectively. Accordingly, the ideal CO_2_/CH_4_ selectivity (the ratio of the two permeabilities) was found as 11.28. This outcome coincided with the good mass of literature reporting the CH_4_-rejective behavior of different polyimides. Typical CO_2_/CH_4_ selectivity data (obtained under mostly varying experimental conditions) were summarized in some articles for a wide range of polyimides, for instance: 16–64 [26] and 13.6–87 [27].

In the next phase of the experiments, the change of gas permeation and separation performance were tested and assessed after various exposures (according to the experimental plan) to pollutants, such as H_2_S, BTX, and dodecane. In all cases, a simple, linear-type association was assumed as a first approach to illustrate the trends in the change of permeability and selectivity using the polyimide membrane.

### 3.1. The Effect of H_2_S Exposure on CO_2_/CH_4_ Separation

Shown in Figure 4, the impact of H_2_S exposure on CO_2_ and CH_4_ permeability can be clearly drawn. From the experimental results, a linear-type correlation seems to be satisfactory to indicate that the contact of the polyimide membrane with higher concentrations of hydrogen sulfide for longer periods caused larger changes in the permeability of the two gases and vice versa. For both gases, it is illustrated in Figure 4 that the permeabilities were increased by the larger H_2_S exposures.

However, considering the ideal selectivity values plotted in Figure 7, the tendency is the opposite compared to those experiences regarding the permeabilities. A significant decrease of CO_2_/CH_4_ separation performance was documented under greater H_2_S exposures. This reverse influence of H_2_S exposure on permeability and selectivity might be explained by the plasticization effect (resulting in the general faster permeation of components and the concurrent drop of selectivity) and/or the modification of the polymer structure. It follows the theory that when H_2_S is present (together with water) in the membrane, it may induce, in some cases, the alteration of inherent material and gas permeation properties [19]. This will have to be further studied in addition to dissecting the reasons why the CO_2_/CH_4_ selectivity could have increased (by 3–4 times) relative to the polyimide unexposed to H_2_S.

### 3.2. The Effect of Dodecane Exposure on CO_2_/CH_4_ Separation

The effect of dodecane exposure on CO_2_ and CH_4_ permeability is demonstrated in Figure 5. It can be inferred, based on the assumed linear relationships (fitted trendlines between the change of exposure and gas permeability), that the larger exposures led to more and more diminished gas permeations through the bunch of polyimide fibers. Concerning the selectivity shown in Figure 8, the tendency of the scattering experimental data reveals no obvious influence, and on average, the CO_2_/CH_4_ selectivity remained quite stable around 9.3 (in accordance with the fitted trendline in Figure 8). Nonetheless, compared to the pristine polyimide (Figure 10), some decline of the CO_2_/CH_4_ selectivity can be noted, and this means that the presence of dodecane had a real effect on the membrane performance.

To provide some plausible explanation, some findings of the relevant literature may be recalled here pertaining to the operational/testing experiences of gas separation membrane technology deployed for natural gas processing. The considerable swelling and, consequently, the change of the separation behavior over time could be concluded for silicone-based membranes upon exposure to heavier hydrocarbons [1,28]. In our opinion, one scenario could have been that the dodecane deposited on the membrane surface and formed a thin (microscopic-scale), fouling-layer like film. This may have automatically reduced the permeabilities of both gases, simply due to the increasing thickness of permeation pathway with greater exposure; however, in total, it did not really modify the separation selectivity.

### 3.3. The Effect of BTX Exposure on CO_2_/CH_4_ Separation

The permeability changes of CO_2_ and CH_4_ gases as a result of different BTX exposures are displayed in Figure 6. As a matter of fact, it can be concluded that the BTX exposure influenced the permeation of both gaseous compounds in a similar manner and the stronger BTX exposures were coupled with the more considerable increase of permeabilities. In terms of CO_2_/CH_4_ selectivity, to the naked eye, the various BTX exposures did not cause apparent changes, as respective values represented by the trend line in Figure 9 consistently spanned the narrow range of 9.4–10. In the literature, the presence of aromatic components, e.g., toluene, was found to impair the separation performance by altering the CO_2_ and CH_4_ permeabilities and depressing the CO_2_/CH_4_ separation selectivity [20]. Similar results were communicated more recently in other investigations [29,30]. Nevertheless, effects associated with toluene could be reversible [31], which is positive from the aspect of membrane stability.

## 4. Conclusions

In this work, the effect of some common natural gas pollutants (hydrogen sulfide, BTX, and dodecane) on the permeability of CO_2_ and CH_4_ gases was studied, applying polyimide hollow-fiber membrane. It was found that all of the investigated pollutants had an impact on the membrane’s performance but in different ways and to different extents. The hydrogen sulfide increased the permeability of both CO_2_ and CH_4_ and the CO_2_/CH_4_ selectivity had a decreasing tendency as a function of increasing H_2_S exposures. In the case of dodecane, permeability of CO_2_ and CH_4_ was decreased moderately by increasing the degree of exposure, while the CO_2_/CH_4_ selectivity, according to tendencies, was left unaffected. By contrast, larger exposures to BTX caused the increase of gas permeabilities; however, the corresponding trends indicated only marginal changes of CO_2_/CH_4_ selectivity. Though possible reasons to explain the dependency of permeability and selectivity on pollutant exposures using the polyimide membrane were implied, further exploration is intended to find out the underlying mechanisms taking place between the actual contaminant and the membrane and to get some insights into whether the observed influences are reversible or irreversible. In future studies, the scope might be expanded to other polymeric membranes, and when a good mass of data are collected, more generalized conclusions may be drawn.

## Figures and Tables

**Table 1:** The experimental boundaries in this work.

Pollutant	C_low_ [ppm]	C_cent_ [ppm]	C_high_ [ppm]	t_1_ [day]	t_2_ [day]	t_3_ [day]
H_2_S	100,000	300,000	500,000	1	3.5	7
BTX	500	750	1000	1	3.5	7
dodecane	1000	5500	10,000	1	3.5	7
